# Multivitamins and cognitive health in older adults: bridging evidence, gaps, and controversies – a comprehensive narrative review

**DOI:** 10.1097/MS9.0000000000004720

**Published:** 2026-01-21

**Authors:** Muhammad Daniyal, Sadia Tameez-ud-din, Muddassir Khalid, Muneeb Faiz, Nouraiz Abbas, Muhammad Hassaan Javaid

**Affiliations:** a Department of Medicine, Bacha Khan Medical College, Mardan, Pakistan; b Department of Medicine, Foundation University Medical College, Islamabad, Pakistan; c Department of Medicine, Nishtar Medical University, Multan, Pakistan; d Department of Medicine, King Edward Medical University, Lahore, Pakistan; e Department of Medicine, ABWA Medical College, Faisalabad, Pakistan; f Department of Medicine, Shifa College of Medicine, Islamabad, Pakistan

**Keywords:** cognitive function, memory, mild cognitive impairment, multivitamins, narrative review, nutritional supplements

## Abstract

**Introduction:**

There is a high prevalence of vitamin deficiency in our healthy population; however, age-related cognitive decline is an emerging global health concern, especially with an aging population and rising cases of dementia. Many trials have been performed to assess the improvement in cognitive functions of people, especially those of older age, by daily multivitamin supplementation. Studies have shown that daily multivitamin intake significantly improves memory in older adults and reduces the risk of cognitive decline.

**Methods:**

A comprehensive search was conducted across PubMed, Embase, and Google Scholar databases from 2003 to 2023. Studies were included based on predefined PICO criteria, focusing on older adults with cognitive decline receiving multivitamin supplementation compared to placebo, with cognitive improvement as the outcome. Nineteen studies meeting the inclusion criteria, comprising randomized trials, reviews, cohort, and cross-sectional designs, were analyzed.

**Results:**

The findings reveal mixed outcomes. Multivitamin supplementation was associated with cognitive improvements, particularly in memory, global cognition, and attention, mainly in individuals with baseline deficiencies or mild cognitive impairment. B-complex vitamins and antioxidant-rich formulas showed the most promising effects. However, in well-nourished or healthy populations, results were often neutral. Variability in study design, supplement composition, assessment tools, and duration were key contributors to these inconsistencies.

**Conclusion:**

The use of multivitamins shows potential cognitive benefits in specific subgroups, particularly the elderly with nutritional deficiencies or early cognitive decline. However, universal cognitive enhancement cannot be concluded. Personalized, targeted supplementation guided by nutritional status may represent the future of cognitive health strategies.

## Introduction

Vitamins are vital micronutrients that cannot be synthesized endogenously or in insufficient amounts, and the principal means by which we get vitamins is through our diet^[1]^. There are 13 essential vitamins – vitamins A, C, D, E, K, and the B vitamins (thiamine, riboflavin, niacin, pantothenic acid, biotin, B_6_, B_12_, and folate)^[2]^. Elderly people are at increased risk for nutrient deficiency, mainly due to diminished food intake with advanced age^[2,3]^. Cognition is the mental action or process of acquiring knowledge and understanding through thought, experience, and the senses. It encompasses various aspects of high-level intellectual functions and processes such as attention, memory, knowledge, decision making, planning, reasoning, judgment, perception, comprehension, language, and visuospatial function, among others. “Cognitive deficit” is used to describe the impairment of different domains of cognition^[4]^. Cognitive health depends upon factors like a healthy diet, physical fitness, social interaction, etc^[5]^. There is a high prevalence of vitamin deficiency in our apparently healthy population^[6]^

Out of these 13 vitamins, mainly Vitamin B12 deficiency has been associated with cognitive impairment^[7]^. Many trials have been performed to assess the improvement in cognitive functions of people, especially in old age, by daily multivitamin supplementation. Studies show that daily multivitamin intake significantly improves memory in older adults and reduces the risk of cognitive decline^[8,9]^. Every day, multivitamin supplementation not only decreased levels of homocysteine but also caused increased levels of vitamin B6 and B12, and both these parameters have a positive impact on the cognitive functions of adults. Multivitamins are also effective in treating depression^[10]^. Multivitamin combined with herbs also enhances the memorizing ability of old people^[11]^. At the cellular level, neural efficacy is enhanced, and memory is improved^[12]^. Multivitamins are not hepatotoxic, so they can be used for prolonged periods of time. Multivitamin alone as well as with mineral and guarana combo improves mood, decision-making performance, enhances the activity in areas associated with working memory, attentional processing, and stable autonomic nervous system function during the first hour^[13,14]^.

All studies didn’t support the fact that daily multivitamin supplementation enhanced memory and treated dementia in old people. As per a few studies, multivitamins had to be combined with herbal, mineral, and guarana to obtain cognition improvement^[11,13,14]^. However, the majority of the studies revealed that there was no difference found in mean cognitive change over time between the multivitamin and placebo groups^[15,16]^. Maybe the doses of vitamins are too low but there was no evidence for a beneficial effect of daily multivitamin and multimineral supplements on memory, knowledge, and learning^[16]^. In one study, a multivitamin showed encouraging effects on one gender but no effects on the other^[17]^. Vitamin B6, B12, folate rise, and homocysteine fall may^[10]^ or may not^[18,19]^ improve cognition. A combination of long-chain omega-3 fish oils or cocoa extract with multivitamins has no cognitive improvement^[20,21]^. Similarly, vitamin B and herbal supplementations may or may not give cognitive benefits due to the phenomenon of “co-nutrient optimization” and interdependency of nutrients^[7,18,19]^. Not even children who were assessed through BSID-III scores gained any cognitive improvement from daily multivitamin intake^[22]^. Antioxidants and B vitamins in combination also have no effect on cognitive performance. Maybe the population was not too well nourished to begin with^[23]^. Daily use of multivitamin supplementation for memory and cognition improvement gave conflicting results. Sometimes it’s effective but in the majority of studies, it’s not. Using combinations has inconsistent results. There is no definite answer to it. There are limitations and contradictory results in previous studies which are required to be evaluated thoroughly. The primary research question guiding this review is: To what extent does multivitamin supplementation improve specific cognitive domains in older adults, particularly those with baseline deficiencies or mild cognitive impairment?” We intend to extract and analyze all previous data and come to a definite conclusion on whether multivitamins are effective in improving the cognitive abilities of older adults or not.

## Methodology

### Literature search

A comprehensive literature search was done on the relation of multivitamins with cognition, across multiple databases including PubMed, Google Scholar, Embase, etc. Specific search terms such as “Multivitamin,” “Supplements,” “Nutritional Supplements,” “Cognition,” and “cognitive health” were used to find relevant studies.

### Study selection

The first author independently reviewed all articles retrieved from the search. Titles, abstracts, and reference lists were screened by the first and second authors against predefined eligibility criteria. To ensure a systematic approach, studies were assessed for inclusion based on the PRISMA framework. After the final search using all the databases, 1289 articles were screened. Around 1172 articles were screened out as they did not fit the criteria. All the articles between 2003 and 2023 were included. After the final screening, 19 articles were selected for data extraction. The articles included in the study are 3 systematic reviews, 1 cross-sectional, 1 prospective cohort, 10 review articles, 3 randomized clinical trials, and 1 quasi-project. The study was designed to be conducted as a systematic review, but it was deferred because not enough randomized clinical trials were available to write the latest systematic review.

**HIGHLIGHTS**

- Multivitamin use shows modest cognitive benefits in older adults with deficiencies.
- Improvements are most consistent in memory, attention, and global cognition.
- Well-nourished or cognitively healthy adults show little to no cognitive benefit.
- Effects vary due to differences in dosage, formulations, and assessment methods.
- Personalized, nutrition-guided supplementation may optimize cognitive outcomes.

### Eligibility criteria

Studies were included based on predefined PICO criteria:

(P) Population: patients with impaired cognitive health.

(I) Intervention or exposure: multivitamin.

(C) Comparison: placebo.

(O) Outcomes: improved cognitive health.

Eligible studies included systematic reviews, review articles, randomized clinical trials, cohort and cross-sectional studies. Only English-language studies were considered. Articles unrelated to cognitive health and multivitamins, non-English studies, and duplicates were excluded.

### Data extraction

**Figure 1.**
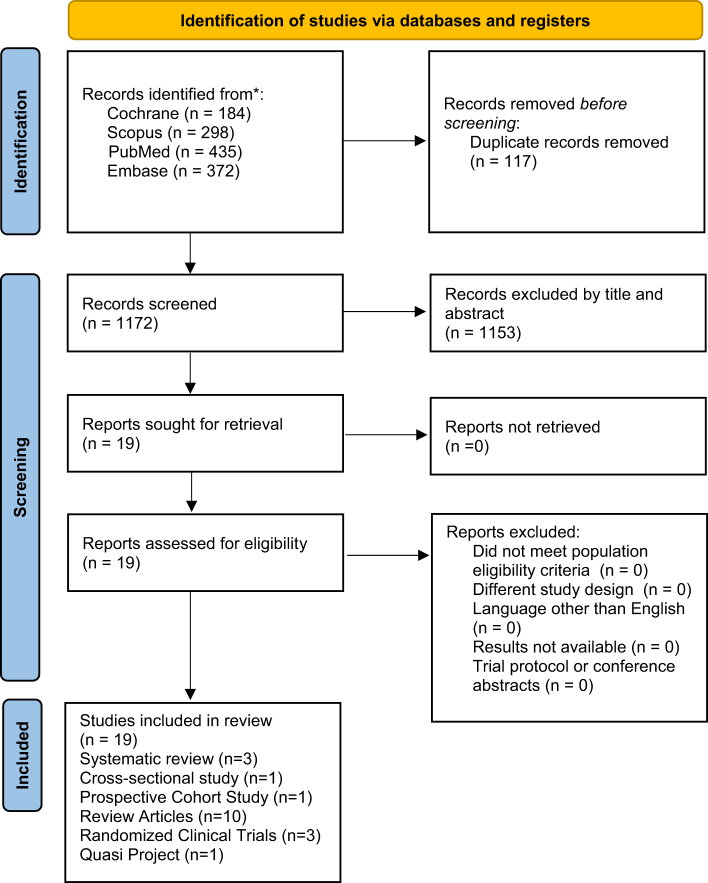
PRISMA flowchart.

Source: Page MJ, *et al*. BMJ 2021;372:n71. doi: 10.1136/bmj.n71. This work is licensed under CC BY 4.0. To view a copy of this license, visit https://creativecommons.org/licenses/by/4.0/

Data extraction was performed independently by the authors using standardized techniques, including Excel spreadsheets for data organization and EndNote for managing references. Extracted data included study characteristics (author, year, and design), patient demographics (age, sex, and ethnicity), intervention details (multivitamin and its effect on cognition), results (outcomes and prognosis), and conclusions. Discrepancies in data extraction were resolved through team discussions to ensure accuracy and consistency. The study selection process, including identification, screening, eligibility assessment, and final inclusion of studies, is detailed in the PRISMA flowchart of Figure 1.

We extracted data including year, age of the participants, placebo vs control, intervention, outcome, and the difference in placebo vs control group results. The data extracted was then used to formulate the results of this study.

### Quality assessment and handling of heterogeneity

Given the narrative nature of this review and the heterogeneity of included study designs, a structured qualitative approach to quality assessment was applied. Randomized controlled trials were evaluated using key elements from the Cochrane Risk of Bias 2 (RoB2) framework, focusing on randomization procedures, blinding, allocation concealment, and outcome reporting. Observational studies were assessed using adapted domains from the Newcastle–Ottawa Scale, including selection, comparability, and outcome ascertainment.

To reconcile methodological disparities, studies were grouped and synthesized according to:

(i) cognitive domain assessed;

(ii) supplement formulation and dosage;

(iii) baseline nutritional or cognitive status of participants.

Because of substantial variation in dose, duration, and cognitive tools used, a meta-analytic approach was not appropriate. Instead, findings were integrated narratively, emphasizing patterns supported across multiple high-quality trials.

## Results

### Overview of multivitamins and cognitive function

Multivitamins used for cognitive health have been widely explored in the older adult population compared to others. And it focused mainly on areas of age-related cognitive decline, mild cognitive decline, or mild cognitive impairment (MCI), and the early stage of dementia. Most of the studies on the relation of multivitamins with cognitive health assessed a combination of vitamins and minerals, intended to support neurological functions, instead of only assessing multivitamins. Randomized controlled trials and reviews included in our study show mixed evidence regarding relation of multivitamins with cognitive health, for example, some studies like Yeung *et al*[24] and Lee *et al*[10] show statistically significant improvement in cognitive health with the use of multivitamins while some studies like Grodstein *et al*[15] shows no notable difference between multivitamin and placebo groups when administered. Among specific domains affected by multivitamins, memory and global cognition appear to be the most consistently improved domains. Furthermore, studies included in our review show that intake of multivitamins is more effective in individuals with baseline nutritional deficiencies or preclinical cognitive symptoms, rather than well-nourished and healthy patients or patients with advanced clinical symptoms. After all, dietary patterns, coexisting health conditions, and formulation differences are key moderating factors about multivitamins relation with cognition.

### Mechanisms of action

Based on the included studies, multivitamins may support cognitive functioning through different interconnected biochemical and physiological mechanisms. Some of the specific mechanisms through which multivitamins can improve cognitive health include the following:

#### Homocysteine regulation

Homocysteine, which is an amino acid and a product of naturally occurring reactions in the body, if elevated, can show neurotoxic effects and is recognized as a significant risk factor for cognitive decline (Zhang *et al*[25]; Olaso-Gonzalez *et al*)[26]. Vitamins B6, B12, and folic acid play an important role in converting homocysteine to methionine; as a result, they lower homocysteine levels in the body, thereby decreasing the neurotoxic effects of homocysteine and thus lowering the risk of cognitive decline. Elevated homocysteine is known to impair endothelial function, reduce methylation capacity needed for neurotransmitter synthesis, and promote oxidative stress and neuroinflammation. By lowering homocysteine, B vitamins indirectly support neuronal integrity, synaptic plasticity, and cerebral perfusion.

#### Antioxidant protection

**Figure 2.**
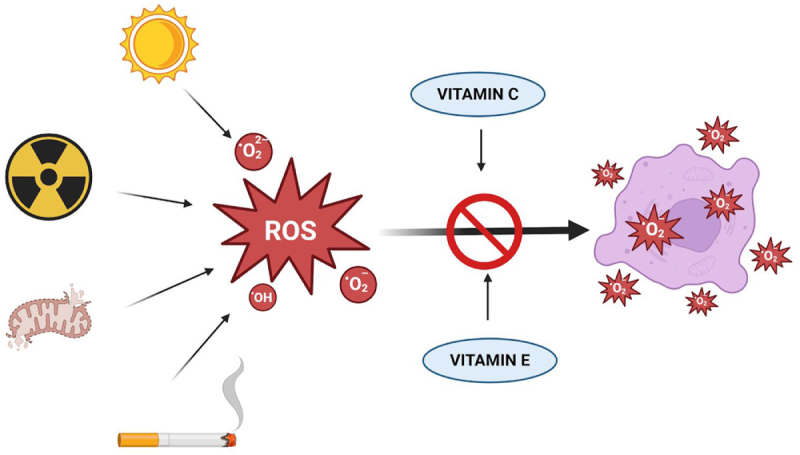
The antioxidant role of vitamin C and vitamin E.

Antioxidants are naturally occurring substances that play an important role in the preservation of neuronal membrane integrity and a reduction in neuro-inflammation by neutralizing the free radicals, thus playing a protective role. Vitamins C and E function as potent antioxidants, helping to neutralize free radicals, thus playing a role in improving cognitive decline (Kennedy)[27] as shown in Figure 2. Antioxidant vitamins also reduce lipid peroxidation of neuronal membranes and attenuate microglial activation, thereby preserving neural signaling pathways essential for memory and executive functioning.

### Specific cognitive domains affected

Based on the included studies, multivitamin supplementation has shown improvements across various cognitive domains like memory, global cognitive function, attention and processing speed, and executive function. But their effects often depend on factors such as formulation, dosage, duration, and the characteristics of the study population.

Evidence suggests domain-specific improvements in memory functions after being measured by specific tools and tests. Episodic memory, which involves recalling personal experience and specific events that occurred in the past, has been shown to improve in long-term trials, as measured by tools like the Modified Rey Auditory Verbal Learning Test (Mod Rey) (Yeung *et al*)[24]. Verbal memory enhancements have been reported using assessments such as the Auditory Verbal Learning Test (AVLT) and the Logical Memory Test (Tao *et al*)[28]. Working memory, which is information related to the task at hand, has shown fair improvements, but is typically observed in specific subgroups rather than across broad populations.

In these studies, multivitamin interventions have shown mixed outcomes in executive function (it is a higher level of cognition that controls and coordinates other cognitive abilities and behaviors). Executive functions like decision-making and cognitive flexibility in individuals with MCI have shown notable improvements (Solfrizzi *et al*)[29]. However, results in healthy populations remain inconsistent, with some studies reporting minimal or no change (Grodstein *et al*)[15].

Multivitamin supplementation, specifically high doses of vitamin B complex, has been shown to increase attentional performance, and improvements in reaction time and attentional control were observed in several studies. Furthermore, in a recent study, the cognitive demand battery also indicated enhanced processing speed and focus in participants receiving supplementation (Fekete *et al*)[30].

Global cognitive functioning which is an overall capacity of the brain to process information and perform various mental tasks, its performance vary depending on baseline status, statistically significant gains in global cognitive scores, including the Mini-Mental State Examination (MMSE) and Alzheimer’s Disease Assessment Scale-Cognitive Subscale (ADAS-Cog) (standard tests used to assess global cognitive functioning), have been noted in elderly individuals with low baseline cognition (Li *et al*)[31]. In contrast, no significant improvements were found in healthy, well-nourished populations (Table 1).

**Table 1:** Specific cognitive domains affected

Cognitive domain	Affected by supplementation?	Assessment tools used	Key findings
Memory	Yes (episodic, verbal, working)	ModRey, AVLT, Logical Memory Test	Consistently improved, especially in MCI
Executive function	Mixed results	Verbal Fluency, ADAS-Cog	Some benefit in at-risk groups; not always significant
Attention and speed	Occasionally improved	Cognitive Demand Battery, Reaction Time Tasks	Reported in high-dose trials, e.g. Kennedy
Global cognition	Frequently improved	MMSE, MoCA, ADAS-Cog	Stronger gains in B-vitamin trials

### Types of nutritional supplements investigated

In our study, different types of nutritional supplements studied for their cognitive effects have been included. They all vary in scope and composition, as well as targeting different neurobiological processes.

Vitamin B-complex has been searched more widely for cognitive support as compared to other vitamins. Their role in decreasing homocysteine levels and their role in brain metabolism have shown promising results in preventing cognitive decline, particularly in older adults (Calderon-Ospina *et al*)[32].

Along with vitamin B complex, other vitamins like D, E, C, and A have also shown benefits in cognitive health. These vitamins contribute to cognitive health through different mechanisms, like neuroprotection, antioxidant activity, and modulation of inflammatory pathways. While having minor effects on the healthy population, their effects are quite remarkable in individuals with deficiencies or elevated oxidative stress (Grima *et al*)[33].

As mentioned, the benefits of antioxidants in cognitive health, some studies have shown that a combination of these antioxidants with Omega-3 fatty acids, particularly EPA and DHA, can improve executive functions and neuronal resilience (Morris *et al*)[34]. Nowadays, omega-3 fatty acids are commonly combined with antioxidant supplements in intervention studies.

Recently, multivitamin and multi-mineral formulations have also been assessed in large cohort studies, with some evidence suggesting fair cognitive benefit in older adults. However, these findings mainly depend on baseline nutritional status and cognitive health (Jittat *et al*)[35] (Table 2).

**Table 2:** Types of nutritional supplements and their cognitive effects

Supplement type	Key nutrients	Mechanisms	Cognitive domains affected	Key studies
B-complex vitamins	B6, B12, folic acid	Homocysteine reduction, neurotransmitter synthesis	Memory, Global cognition	Zhang *et al*[25]; Lee *et al*[10]
Fat-soluble vitamins	Vitamin D, E, A	Neuroprotection, antioxidant defense, neurotrophin regulation	Executive function, Global cognition	Kennedy[27]
Vitamin C	Ascorbic acid	Antioxidant, synergistic with vitamin E	Attention, Processing speed	
Omega-3 fatty acids and antioxidants	DHA, EPA, polyphenols, flavonoids	Anti-inflammatory, neuroplasticity support	Executive function, Memory, Speed	Tucker[41]
Multivitamin/mineral formulas	Centrum Silver, multi-nutrient blends	Multiple – synergistic roles in energy, protection, signaling	Memory, Global cognition (varied results)	Yeung *et al*[24]; Grodstein *et al*[15]

### Effect of duration and dosage

The efficacy of multivitamin intake in cognition is mainly dependent on the duration and dosage of intervention, but considerable heterogeneity exists across studies.

Intake of multivitamins for short-term (4 weeks–6 months) has shown early improvements in mood and certain cognitive functions, specifically in those who are at risk of its deficiency or who have started cognitive decline (Macpherson *et al*)[36].

Longer trials, which may vary from 6 months to 14 years, have shown mixed results. Across studies, dosage standardization was a major methodological limitation. Some interventions used RDA-level multivitamins while others employed high-dose or multi-component formulations combining vitamins, minerals, and herbal agents. This inconsistency in dosing makes cross-study comparisons challenging and likely contributes to the mixed cognitive outcomes observed. Some show constant improvements in cognitive health with proper adherence, while others find little benefit over time, which suggests the need for consistent adherence to multivitamin intake and appropriate baseline targeting (Vyas *et al*)[37].

### Population demographics and moderating variables

According to the present literature, the efficacy of multivitamin supplementation and its impact on cognitive health is not uniform across all individuals and is largely affected by demographic and physiological variables.

Compared to the well-nourished population and having healthy cognition, older adults with MCI tend to experience greater cognitive benefits from multivitamin supplementation. This suggests that multivitamin supplementation may be most effective in populations already experiencing cognitive decline as compared to healthy populations (Grima *et al*)[38].

Multivitamin effects on cognition, comparing genders, have limited literature, and are not studied in much detail, however, some studies suggest that multivitamin intake can be more beneficial in men as compared to women, if every other demographic is kept the same. This probably can be due to metabolic or hormonal differences.

Baseline nutritional deficiency is also an important variable, and as explained in detail, baseline nutritional deficiencies or chronic illness can make multivitamins’ positive effects more amplified. This explanation is supported by this study, which says individuals who are malnourished or have elevated homocysteine levels will show the greatest improvement in cognitive outcomes (Gutierrez *et al*)[39].

Furthermore, Tao *et al*[8] suggest that multivitamins can work best if combined with other health-promoting behaviors, such as regular physical activity and mental engagement (e.g. reading), etc.

### Cognitive assessment tools and tests used across studies

Different types of standardized cognitive tools and tests have been used to measure outcomes in supplementation in different studies. Such as MMSE, this tool is used most commonly and is used to assess global cognitive functions. MoCA (Montreal Cognitive Assessment) is a more sensitive assessment tool specifically used to detect early cognitive changes, along with that it covers many specific domains. ADAS-Cog (Alzheimer’s Disease Assessment Scale–Cognitive Subscale), this assessment tool is frequently and specifically used in Alzheimer’s and dementia trials. Furthermore, some domain-specific tests like ModRey, AVLT, Logical Memory, Digit Span, Verbal Fluency, have been used. These domain-specific tests assess episodic memory, working memory, verbal ability, and executive functioning. The selection of tests can sometimes influence the detected outcomes and may contribute to inconsistencies across studies. The heterogeneity of cognitive tests across studies also limits comparability. Tools such as MMSE and MoCA differ significantly in sensitivity, particularly for early cognitive changes, while domain-specific tests (e.g. ModRey, AVLT, Digit Span) further introduce variability based on which cognitive domains are prioritized. These methodological inconsistencies may partially explain why some trials report cognitive improvement while others do not.

### Efficacy and study limitations

Despite growing interest of new researchers in relation to cognition with nutritional supplementation, and an immense amount of literature on this topic, findings remain mixed and influenced by methodological variability.

As suggested by many studies, improvements are most consistently observed in global cognition function and memory-related domains, specifically among older adults and those who are already suffering cognitive decline. But impacts on other specific domains like executive functioning and attention remain inconsistent across studies.

There is some part of selection bias, over the selection of studies, as randomized controlled trials are more core knowledge of this review compared to observational studies, and as observational studies tend to report more optimistic outcomes compared to placebo-controlled randomized controlled trials, which usually show more modest or null effects.

Other key limitations include heterogeneous test batteries, underrepresentation of diverse demographic groups, and relatively short follow-up durations. These factors reduce the generalizability and reproducibility of study findings.

### Safety and interactions

Multivitamins are generally safe when appropriately dosed, but some points should be considered regarding their safety and interactions before considering them.

High-dose intake of multivitamins, especially if taken with fat-soluble vitamins like vitamin A and minerals like iron, carries a high risk of toxicity. Based on this study, over-supplementation can result in adverse effects, including liver damage or gastrointestinal distress (Vanessa Pike *et al*)[40].

**Figure 3.**
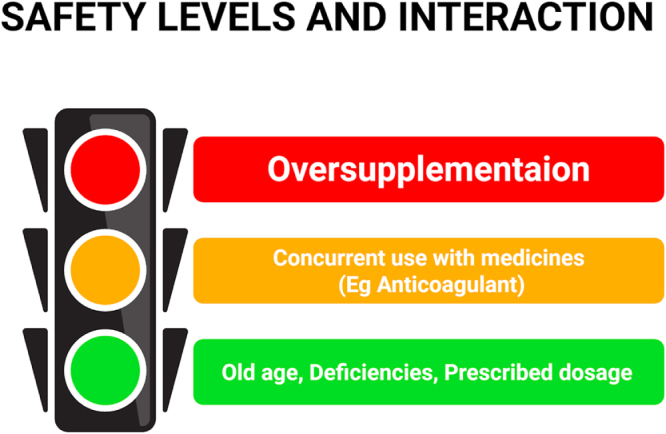
Safety levels and interactions.

Administering supplements or multivitamins to individuals with their deficiencies, old age, and comorbid conditions improves both safety and efficacy of multivitamins. Multivitamins or supplements should be used carefully when using medications like anticoagulants, as they may interact with medications (e.g. anticoagulants) or with specific dietary components, and can cause serious side effects (Fig. 3).

### Future directions and research gaps

Covering immense literature and after getting valuable findings, however there are still several gaps that remain in the current understanding of how and for whom nutritional supplementation benefits cognition.

**Figure 4.**
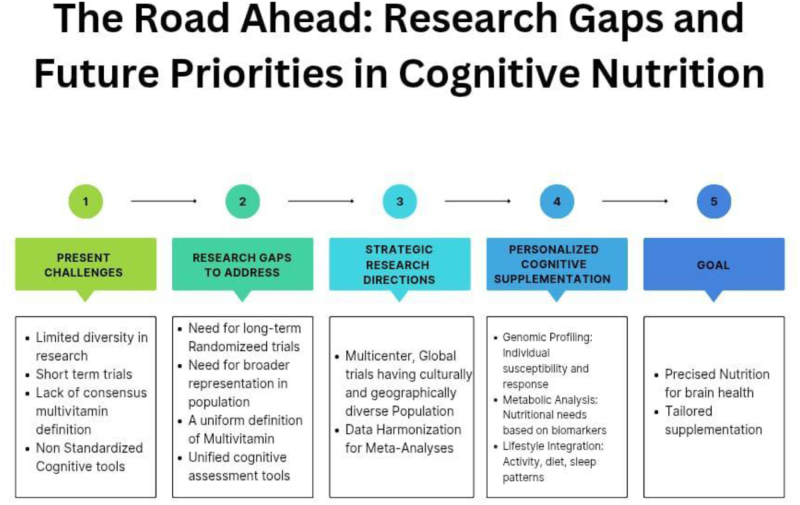
Illustration showing future aspects and research gap.

There is a dire need for long-term, rigorously controlled trials involving diverse populations, including underrepresented ethnic, gender, and socioeconomic groups. Broader representation will enhance the relevance of findings to the general population. A uniform definition of “multivitamin” and standardization of cognitive assessment tools are needed to facilitate data comparison and meta-analyses across studies. The future of cognitive supplementation likely lies in personalized nutrition, informed by genomic, metabolic, and lifestyle profiling. This precision approach may optimize outcomes and reduce risks associated with indiscriminate supplementation (Tucker)[41] (Fig. 4).

## Conclusion

The results from trials available so far indicate that multivitamin supplementation is associated with modest improvement in cognition, especially in memory, attention, and global cognition in a selected population of older adults with nutritional deficiencies or mild cognitive impairment. Such changes likely occur through homocysteine regulation and antioxidant protection. However, the findings actually vary widely among studies, with several trials showing no significant improvement in either well-nourished or cognitively healthy populations. Such variability can be explained by differences in supplement formulation and dosage, study design, and assessment tools.

In summary, this would seem to suggest that multivitamins may have a supportive role in selected individuals rather than as an intervention across the board. A personalized approach is thus suggested, guided by baseline nutritional status in concert with healthy lifestyle practices. The heterogeneity in supplement formulation, dosage, and cognitive assessment methods underscores the need for rigorous standardization in future research. Further research needs to be directed at standardized formulations, longer follow-up, and targeted populations in order to clarify the true cognitive potential of multivitamin supplementation in aging adults.

## Limitations

Limitations of this review include the heterogeneity between the studies regarding design, sample size, composition of supplement, and assessment of cognition. Most of these trials have a small follow-up duration, whereby long-term effects cannot be ascertained with certainty. Nutritional status at baseline has not been uniformly measured and may have affected the results. Additionally, lack of dosage standardization and variability in the selection and sensitivity of cognitive assessment tools represent important limitations. These inconsistencies reduce cross-study comparability and may have influenced the mixed results reported across trials. Finally, in spite of the comprehensive search, selection bias cannot be entirely ruled out since this is a narrative rather than a systematic review.

TITAN Guidelines: This manuscript is in compliance with TITAN Guidelines, 2025, declaring no use of AI (47).

## Data Availability

Not applicable.
